# Cancer-associated fibroblast-derived gene signatures predict radiotherapeutic survival in prostate cancer patients

**DOI:** 10.1186/s12967-022-03656-5

**Published:** 2022-10-04

**Authors:** Ran Zhang, Feng Liu

**Affiliations:** 1 Laboratory of Radio-Immunology, Shandong Provincial Key Laboratory of Radiation Oncology, Cancer Research Center, Shandong Cancer Hospital and Institute, Shandong First Medical University and Shandong Academy of Medical Sciences, Jinan, 250117 Shandong People’s Republic of China; 2 Department of Immunology, School of Biomedical Sciences, Shandong University, Jinan, 250012 Shandong People’s Republic of China

**Keywords:** Prostate cancer, Radiotherapy prognosis, Cancer-associated fibroblasts, Gene signature, Biochemical recurrence-free survival, Metastasis-free survival

## Abstract

**Background:**

Cancer-associated fibroblasts (CAFs) play multiple roles in regulating tumor metastasis and treatment response. Current clinical indicators are insufficient to accurately assess disease risk and radiotherapy response, emphasizing the urgent need for additional molecular prognostic markers.

**Methods:**

In order to investigate CAF-related genes associated with radiotherapy and construct prognostic CAF-related gene signatures for prostate cancer, we firstly established a radio-resistant prostate CAF cell subline (referred to as CAFR) from Mus-CAF (referred to as CAF) through fractionated irradiation using X-rays. Transcriptome sequencing for CAF and CAFR was conducted, and 2626 CAF-related differentially expressed genes (DEGs) associated with radiotherapy were identified. Human homologous genes of mouse CAF-related DEGs were then obtained.

**Results:**

Functional enrichment analysis revealed that these CAF-related DEGs were significantly enriched ECM- and immune-related functions and pathways. Based on GSE116918 dataset, 186 CAF-related DEGs were correlated with biochemical recurrence-free survival (BCRFS) of prostate cancer patients, 16 of which were selected to construct a BCRFS-related CAF signature, such as *ACPP*, *THBS2*, and *KCTD14*; 142 CAF-related DEGs were correlated with metastasis-free survival (MFS), 16 of which were used to construct a MFS-related CAF signature, such as *HOPX*, *TMEM132A*, and *ZNF467*. Both Gene Expression Omnibus (GEO) and The Cancer Genome Atlas (TCGA) datasets confirmed that the two CAF signatures accurately predicted BCRFS and MFS of prostate cancer patients. The risk scores were higher in patients with higher gleason grades and higher clinical T stages. Moreover, the BCRFS-related CAF signature was an independent prognostic factor and a nomogram consisting of BCRFS-related CAF signature and various clinical factors accurately predicted 2-, 3-, and 5-year survival time of prostate cancer patients. Furthermore, the risk score was positively correlated with multiple immune checkpoints.

**Conclusions:**

Our established CAF signatures could accurately predict BCRFS and MFS in prostate cancer patients undergoing radiotherapy.

**Supplementary Information:**

The online version contains supplementary material available at 10.1186/s12967-022-03656-5.

## Background

Prostate cancer is a common malignancy in male adults worldwide, with more than 1.2 million newly diagnosed cases and 350,000 deaths in 2018 [1]. Radical prostatectomy and radiotherapy are standard therapies for clinically localized prostate cancer. However, biochemical recurrence (BCR) occurs in approximately 20–30% of prostate cancer patients after initial treatment, and may contribute to develop an advanced stage known as castration-resistant prostate cancer (CRPC), leading to the elevated risks of metastasis and death [2–5]. Therefore, elucidation of key molecular mechanism underlying prostate cancer progression and development of novel signatures for predicting radiotherapy outcome and survival will help to improve the management of this malignancy.

Cancer-associated fibroblasts (CAFs) are one of the most dominant components in the tumor stroma, which build up and remodel the extracellular matrix (ECM) structure [6]. As one of the major constituents of the tumor microenvironment (TME), CAFs play multiple roles in regulating tumorigenesis, tumor metastasis, and therapeutic resistance [7, 8]. CAFs are also implicated in the regulation of immune evasion and poor responses to cancer immunotherapy via modulation of many components of the immune system [9]. CAFs-related genes, such as *CALD1* can promote bladder cancer progression by modulating the immunosuppression status of TME and may serve as a prognostic biomarker in bladder cancer [10]. Loss of the membrane protein caveolin-1 in CAFs is associated with radiation resistance of prostate cancer cells and thus affects disease prognosis [11, 12]. Considering that disease recurrence remains high after initial radiotherapy, it is still urgent to explore the important CAF-related genes associated with treatment outcome and disease relapse, which will serve as valuable prognostic biomarkers for patient with prostate cancer.

Herein, we constructed a cell subline resistant to irradiation, named CAFR by X-ray irradiation for Mus-CAF and found that the subline CAFR was more radio-resistant to irradiation than the parental cell line CAF. We then performed transcriptome sequencing for CAF and CAFR to identify CAF-related differentially expressed genes (DEGs) associated with radiotherapy in metastatic prostate cancer. Human homologous genes of mouse CAF-related DEGs were obtained for functional enrichment analysis. Next, we constructed and evaluated the prognostic CAF-related gene signatures that could predict the biochemical recurrence-free survival (BCRFS) or metastasis-free survival (MFS) by combining the public datasets from Gene Expression Omnibus (GEO) database and The Cancer Genome Atlas (TCGA). Furthermore, we evaluated the association between BCRFS-related CAF signature and clinical features, BCR, metastasis (MET) or immune checkpoints. Our efforts will provide a new perspective on the clinical significance of CAF-related genes and help to predict the clinical outcomes of patient with prostate cancer.

## Methods

### Cell line and cell culture

Mus-CAF cell was prepared as a primary culture from 36 weeks-old TRAMP mice and immortalized by SV40 large T-antigen. The origin of this cell line has been described in detail in previous articles [13, 14]. These cells were cultured in Dulbecco's modified Eagle's medium (DMEM) (Sigma–Aldrich, USA) supplemented with 100 U/ml penicillin/streptomycin (P/S, Invitrogen, USA) and 10% fetal bovine serum (FBS, Invitrogen).

### Establishment of radio-resistant cell line

The method for establishing radio-resistant cell line by fractionated irradiation has been described previously [15, 16]. Briefly, the Mus-CAF cell was irradiated with 10 Gy of X-ray irradiation, from a linear accelerator (6-MV X-ray), at a rate of 3 Gy/min when it was first grown to approximately 60% confluence in 25 cm^2^ culture flasks. After reaching approximately 60% confluence, the cell was irradiated with 10 Gy of X-ray for the second time. The fractionated irradiations were continued until the total concentration reaching 80 Gy. The 8 × 10 Gy to generate the radio-resistant cells was according to both the median lethal radiation dose of the cell and the actual situation of clinical application. The radio-resistant cell subline Mus-CAFR was then established. The parental cells were subjected to identical trypsinization, replating, and culture conditions, but were not irradiated. For all assays on irradiated cells, there was at least a 4-week interval between the last 10 Gy fractionated irradiation and the experiment.

### Clonogenic assay

Appropriate numbers of cells (1000, 1200, 1400, 1600, 1800 or 2000/well) were seeded into 6-well plates depending on the different radiation doses and exposed to 0, 2, 4, 6, 8 or 10 Gy radiation respectively. Cells were further allowed to grow for 14 days to form clusters, followed by 4% paraformaldehyde fixation and crystal violet (G1014, Servicebio, Wuhan, China) staining. Colonies comprising more than 50 cells were counted. The calculation formulae for survival fraction (SF): SF = (number of colonies counted / number of colonies seeded) test / (number of colonies counted / number of colonies seeded) control, where “test” denotes the test condition (some radiation dose) and “control” denotes identical cells without radiation.

### Western blot

CAF and CAFR cells were lysed in RIPA lysis buffer to extract the total protein. Protein was quantified using the bicinchoninic acid method. Equal amounts of protein were separated by SDS-PAGE and then transferred onto a polyvinyl difluoride membrane (Millipore, Bedford, MA). The membranes were incubated with primary antibodies overnight at 4 °C. Proteins were detected by appropriate secondary antibodies conjugated with horseradish peroxidase (Bio-Rad, Richmond, CA, USA), followed by enhanced chemiluminescence detection (Pierce, Rockford, IL, USA).

### Detection of apoptotic cells

Cells were washed with PBS and harvested by trypsin without EDTA after 72 h being irradiated. Cells were labeled with an Annexin V-FITC Cell Apoptosis Detection Kit (BD Biosciences, USA) and analyzed by flow cytometry. Apoptosis was evaluated using the Annexin V-FITC Apoptosis Detection Kit (BD Biosciences, USA) followed by FACS analysis.

### β-galactosidase assay

Mus-CAF and Mus-CAFR were seeded at a density of 20.000 cells per well in six-well plates and left for attachment and spreading for 24 h before irradiation. 72 h post-irradiation, cultures were washed and fixed for 5–7 min at 20 ℃ with paraformaldehyde (2%) and stained for β-galactosidase (5-bromo-4chloro-3-indolyl-B-D-galactopyranoside). Staining was achieved following instructions from the manufacturer;“Senescence β-galactosidase Staining Kit”(# C0602, Beyotime). Randomly selected fields were photographed at 100 × magnification.

### Cell proliferation assay

Cell proliferation was assessed using the 3-(4,5-dimethylthiazol-2-yl)-2,5- diphenyltetrazolium bromide (MTT) assay. Briefly, Mus-CAF or Mus-CAFR cells (2 × 10^3^/well) were seeded into 96-well plates. After 0, 24, 48, and 72 h, the medium was replaced with 100 mL of MTT solution (0.5 mg/mL in cell culture medium) and incubated at 37 °C for 2 h. MTT solution was then removed, and MTT formazan was dissolved in 100 mL DMSO. Absorbance was measured at 570 nm.

### Data sources and preprocessing

CAF and CAFR cells were sent to Personalbio Inc. (Shanghai, China) for library construction and next-generation sequencing (NGS). All constructed libraries were sequenced as 150 bp paired-end on a full run (2 × 150 PE) using the Illumina platform. The raw data were then subjected to data filtering. The adapter sequences at the 3’ end were removed by Cutadapt [17] and the reads with average quality lower than Q20 were excluded. The clean reads were mapped to the reference genome using HISAT2 software [18] with default parameters. The read counts were calculated using HTSeq-count [19] and normalized to fragments per kilobase of transcripts per million mapped reads (FPKM).

The microarray data GSE116918 and GSE70769 were downloaded from Gene Expression Omnibus (GEO, https://www.ncbi.nlm.nih.gov/geo/) repository based on the platform of GPL25318 [ADXPCv1a520642] Almac Diagnostics Prostate Disease Specific Array (DSA) and GPL10558 Illumina HumanHT-12 V4.0 expression beadchip, respectively. GSE116918 dataset contained 248 primary prostate cancer tissue samples, 56 of which experienced BCR and 22 of which developed MET. GSE70769 dataset contained 94 primary prostate cancer tissue samples, 45 of which had BCR. Data preprocessing was then conducted. The average value of different probes corresponding to one gene was used as the final expression value of this gene.

The RNA-seq data and clinical data from TCGA Prostate Adenocarcinoma (PRAD) dataset were downloaded from UCSC Xena (http://xena.ucsc.edu) [20]. This dataset contained 484 prostate cancer samples, among which 98 samples had BCR.

### Identification of DEGs

Based on our transcriptome sequencing data, differential expression analysis was performed using the DEseq2 package [21] in R. Genes with less than 10 reads in each row of our transcriptome sequencing dataset were eliminated. The p value was adjusted using the Benjamini–Hochberg procedure. The DEGs between CAFR and CAF groups were identified with threshold value of adj.p value < 0.05 and |log fold change (FC)|> 2. The volcano plot and heatmap for DEGs were created using ggplot2 and pheatmap, respectively.

### Homologous gene transformation in human and mouse and functional enrichment analysis

DEGs were transformed into human homologous genes by biomaRt in R package. Then, Gene Ontology (GO) [22] and Kyoto Encyclopedia of Genes and Genomes (KEGG) pathway [23] enrichment analyses were conducted by Clusterprofiler package [24]. GO function mainly includes three categories, including biological process (BP), cellular component (CC), and molecular function (MF). The p value was adjusted using the Benjamini–Hochberg procedure. The adj.p value < 0.05 was selected as the threshold value.

### Construction of prognostic CAF-related gene signatures

To identify prognostic CAF-related DEGs associated with BCRFS, univariate Cox regression analysis was carried out using survival package in R based on the clinical data in the GSE116918 dataset. The p value and hazard ratio (HR) of each variable was calculated to identify risk genes (HR > 1) and protective genes (HR < 1). Prognostic CAF-related DEGs were obtained with p < 0.01. To minimize overfitting risk, the least absolute shrinkage and selection operator (LASSO) Cox regression analysis was performed using glmnet package [25] in R. The final lambda (λ) for construction of BCRFS-related CAF signature was determined by ten-fold cross-validation. In addition, identification of prognostic CAF-related DEGs associated with MFS and construction of MFS-related CAF signature was performed using the same methods.

### Evaluation of the prognostic CAF-related gene signatures

The risk scores of two CAF signatures were respectively calculated based on the expression level of each gene and corresponding regression coefficients. The optimal cut-off for the risk score was determined using the Survminer R package. Patients were divided into high- and low-risk groups based on the optimal cut-off, followed by analysis of survival difference between the two groups. Moreover, the 1-, 2-, and 3-year prognostic prediction power of two CAF signatures were analyzed by TimeROC package in R. Furthermore, because only BCR data were included in the TCGA and GSE70769 datasets, only the predictive value of BCRFS-related CAF signature was validated using these two external datasets.

### Association analyses of BCRFS-related CAF signature with various clinical factors

Based on the clinical data in the GSE116918 and TCGA datasets, the association of BCRFS-related CAF signature with various clinical factors including clinical T-stage, gleason grade, and prostate specific antigen (PSA) was analyzed using ggstatsplotR software.

### Analysis of the independent prognostic factors and establishment of a nomogram

Based on clinical data in the GSE116918 and TCGA datasets, univariate and multivariate Cox regression analyses were conducted to determine the independent prognostic factors, by analyzing the risk score of BCRFS-related CAF signature and clinical variables, including clinical T-stage, gleason grade, and PSA. The p < 0.05 indicated a significant result. A nomogram was then constructed to predict the 2-, 3-, and 5-year survival probability of patients with prostate cancer.

### Association analysis of genes in CAF signatures with BCR, MET and immune checkpoints

Based on the gene expression data in GSE116918 dataset, gstatsplotR software was used to compare the expression levels of three genes in the BCRFS-related CAF signature, such as *ACPP*, *KCTD14*, and *THBS2* between BCR and non-BCR groups using gstatsplotR software, as well as to analyze the expression of three selected genes in the MFS-related CAF signature, such as *HOPX*, *ZNF467*, and *TMEM132A* between MET and non-MET groups. Sankey diagram was created to display the clinical features associated with BCR and MET. Moreover, the correlation between risk score and multiple immune checkpoints was analyzed.

## Results

### Establishment of cell subline resistant to irradiation

**Fig. 1:**
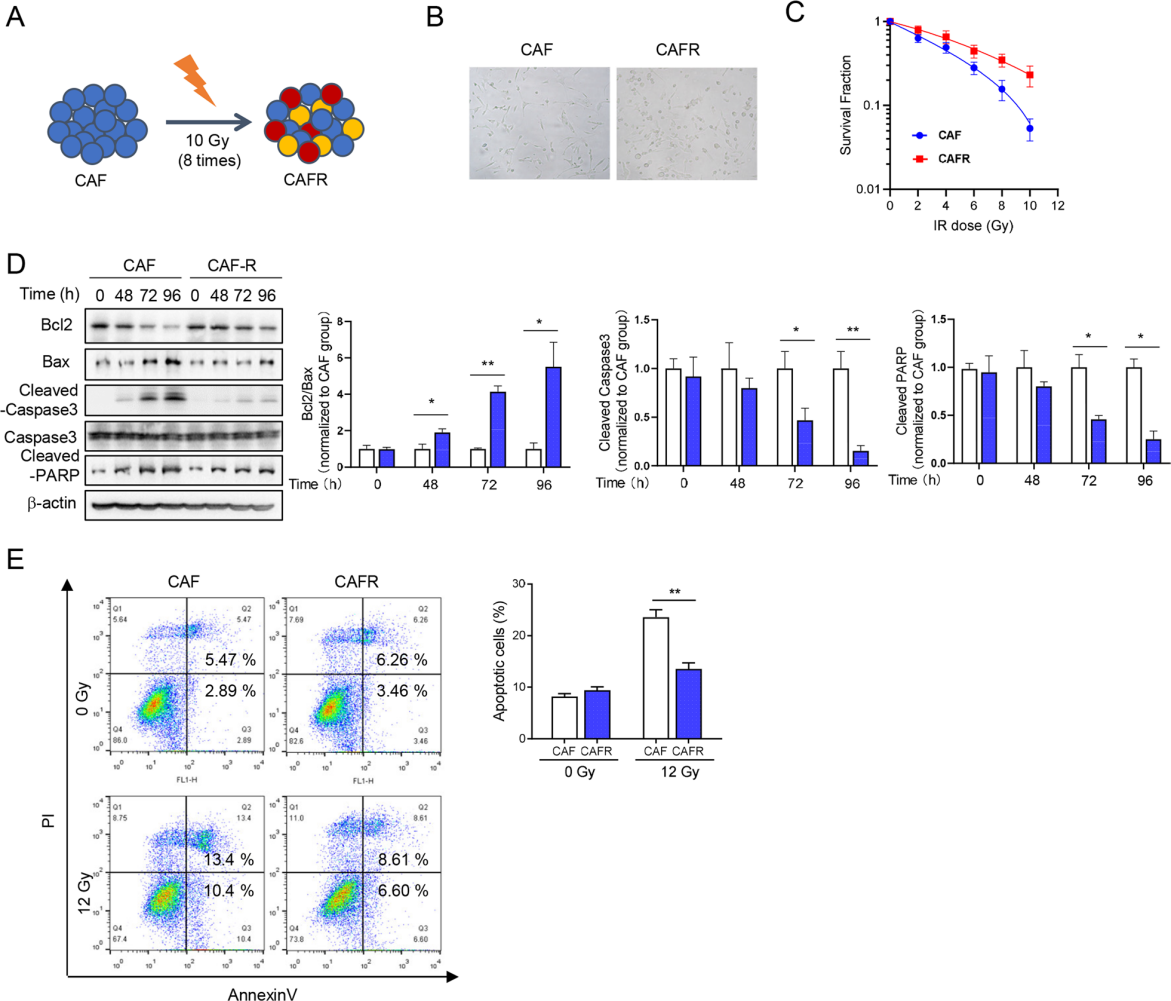
Establishment and identification of a radio-resistant cell subline, named CARF. **A** Schematic diagram shows the construction of CAFR. **B** The cell morphology of CAFR and CAF. **C** Radiation cell survival curves for CAF and CAFR. **D** Expression of the apoptosis-related proteins in CAF and CAFR at different time points detected by western blot after X-Ray irradiation (12 Gy). The band density was quantified using Image J software and normalized to corresponding CAF group. **E** Irradiation-induced apoptosis in CAF and CAFR cells by flow cytometry analysis (12 Gy). The percentage of apoptotic cells was counted. Similar results were obtained in three independent experiments. Errors bar represent the standard error of the mean (*, *p* < 0.05; **, *p* < 0.01; ***, *p* < 0.001)

The Mus-CAF cells were treated repetitively with 10 Gy of X-ray irradiation, with about 7 days recovery allowed between each fraction, until the total dose reaching 80 Gy. The radio-resistant cells were named CAFR (Fig. 1A). We did microscopic observation of cell morphology and found that the CAFR exhibited obvious morphological changes compared with parental cells. A large portion of CAFR showed irregular morphology. Some of them were shorter spindle-shaped, and some cells became more round than parental cells (Fig. 1B). Then the clonogenic assay was performed to analyze their radiosensitivity after 0–12 Gy irradiation and the survival curves showed that the subline CAFR was more radio-resistant to irradiation than the parental cell line CAF (Additional file 1: Figure S1A and Fig. 1C). The senescence and proliferative capacity of CAF and CAFR were also detected through β-galactosidase and MTT assay respectively, and the data exhibited no significant difference between the CAR and CAFR group (Additional file 1: Figure S2A, B). The expression levels of apoptosis-related proteins were detected at different time points after irradiation, and the results showed that the expressions levels of pro-apoptotic proteins BAX, Cleaved PARP and Cleaved Caspase 3 were decreased, while the anti-apoptotic protein Bcl2 was significantly increased in CAFR than CAF (Fig. 1D). The apoptosis induced by 12 Gy irradiation was detected and a significant difference was recognized between CAF and CAFR. The acquirement of radio-resistance was reflected in a reduced apoptotic rate in CAFR compared to CAF (Fig. 1E). These data indicated that CAFR had better radiotherapy resistance and tolerance than CAF and prove that the cell subline resistant to irradiation, named CAFR, was successfully constructed.

### DEGs screening

**Fig. 2:**
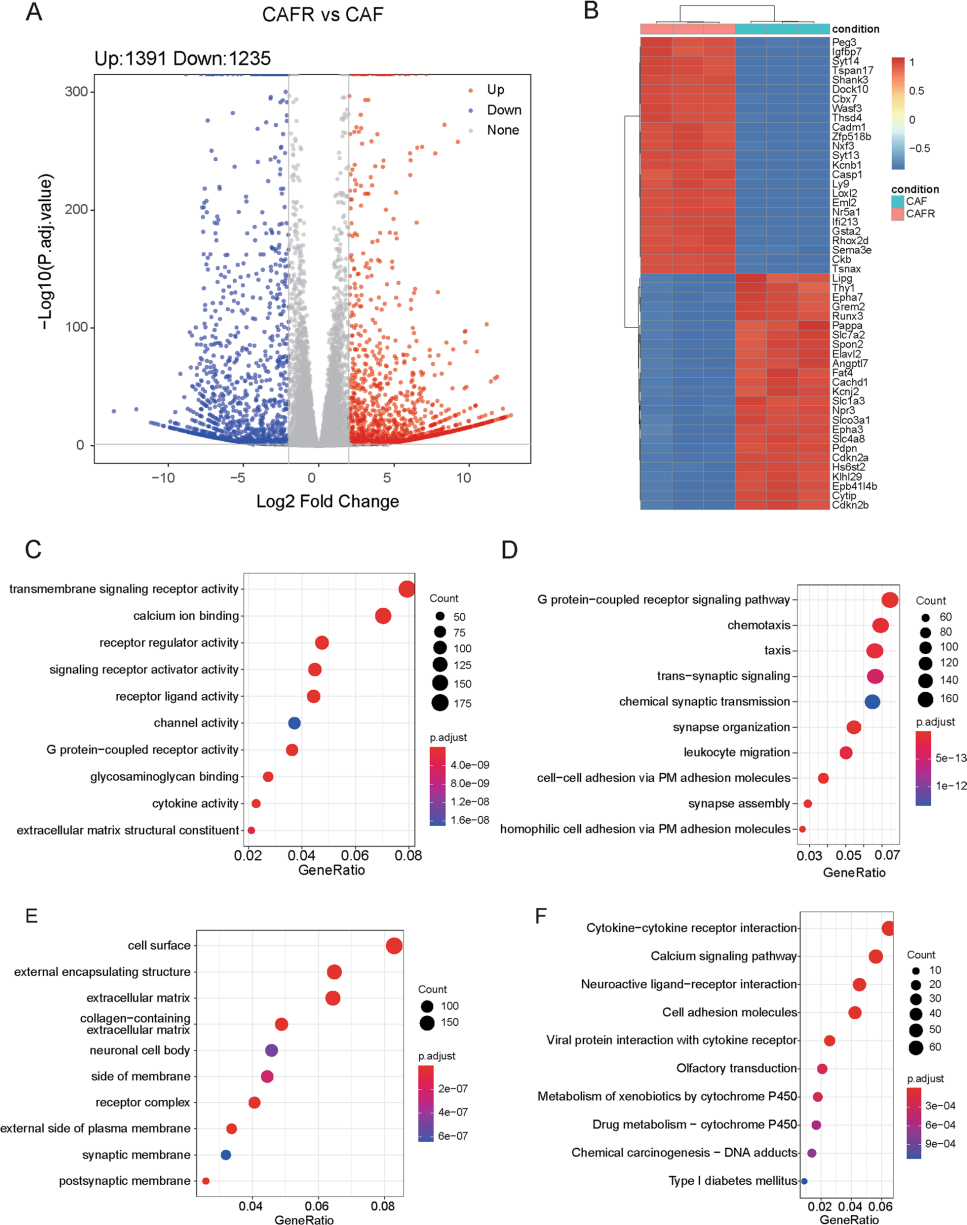
Identification of CAF-related DEGs associated with radiotherapy based on our transcriptome sequencing data and functional enrichment analysis. **A** Volcano plot of DEGs. **B** The heatmap for DEGs. **C** GO-MF terms enriched by DEGs. **D** GO-BP terms enriched by DEGs. **E** GO-CC terms enriched by DEG. **F** KEGG pathways enriched by DEGs. *CAF* cancer-associated fibroblast, *DEGs* Differentially expressed genes, *GO* Gene Ontology, *MF* molecular function, *CC* Cellular component. *BP* Biological process, *KEGG* Kyoto Encyclopedia of Genes and Genomes

In order to clarify the different gene expression profiles between CAFR and CAF, we performed transcriptome sequencing. Based on our transcriptome sequencing data, a total of 2626 DEGs were identified between CAFR and CAF groups including 1391 up-regulated genes and 1235 down-regulated genes (Fig. 2A). The heatmap for DEGs revealed that DEGs could distinguish CAFR samples from CAF samples (Fig. 2B). Human homologous genes of mouse CAF-related DEGs were obtained for functional enrichment analysis. As results, DEGs were significantly enriched in GO MF terms, such as transmembrane signaling receptor activity and extracellular matrix structural constituent (Fig. 2C); GO-BP terms, such as G protein-coupled receptor signaling pathway and regulation of leukocyte migration (Fig. 2D); GO CC terms, such as cell surface and extracellular matrix (Fig. 2E); and KEGG pathways, such as cytokine-cytokine receptor interaction and cell adhesion molecules (Fig. 2F).

### Identification of prognostic CAF-related genes

**Fig. 3:**
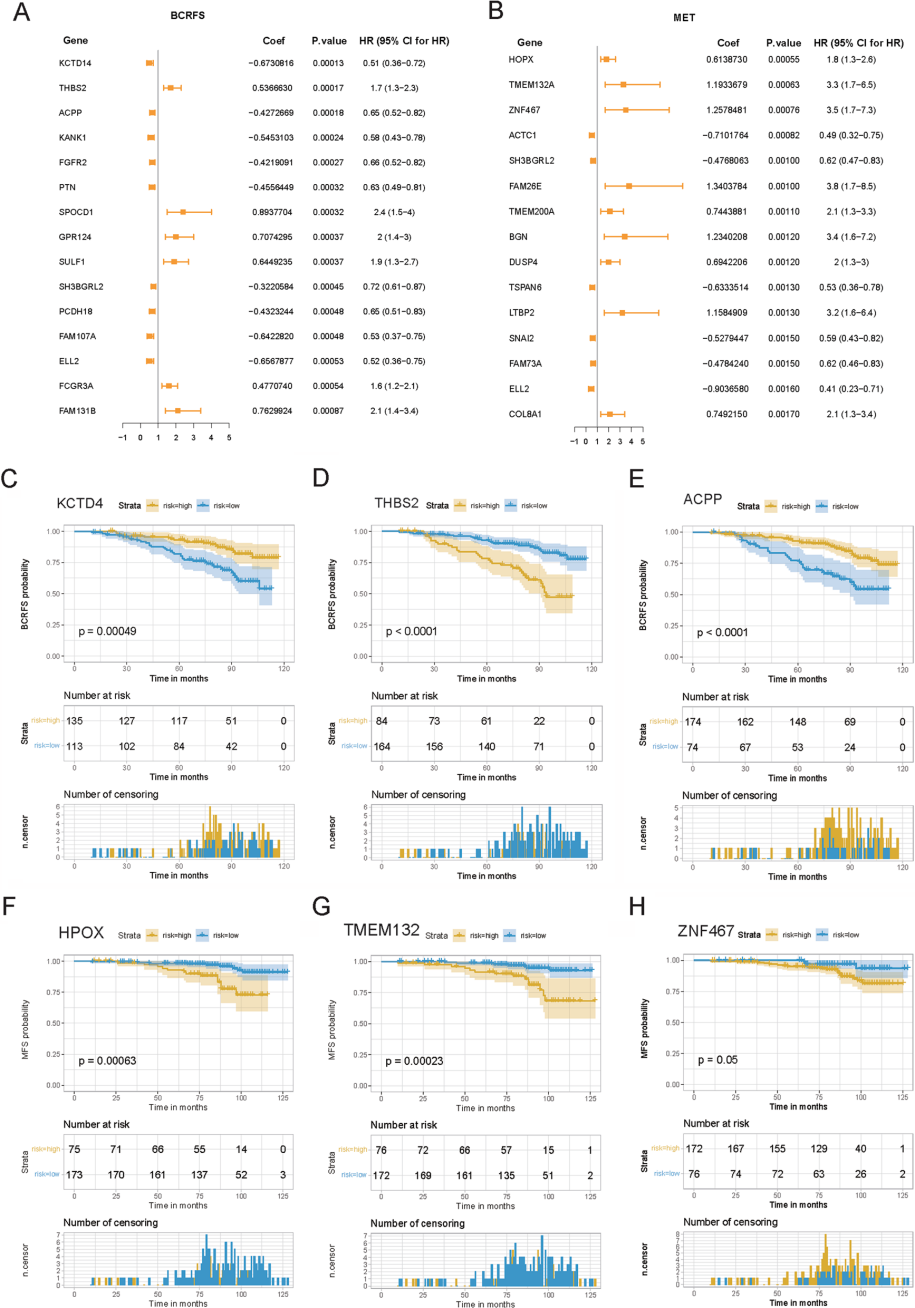
Identification of prognostic CAF-related DEGs based on the clinical data and gene expression data in the GSE116918 dataset. **A**, **B** Forest plot showed the top 15 significant CAF-related DEGs associated with BCRFS or MFS. **C**–**E** Kaplan–Meier survival curve analysis of the top 3 CAF-related genes associated with BCRFS, named *KCTD4*, *THBS2*, and *ACPP*. **F**–**H**: Kaplan–Meier survival curve analysis of the top 3 CAF-related genes associated with MFS, named *HOPX*, *TMEM132A*, and *ZNF467*. *CAF* Cancer-associated fibroblast, *DEGs* Differentially expressed genes; *BCRFS* Biochemical recurrence-free survival, *MFS* Biochemical recurrence-free survival

CAF-related DEGs were transformed into human homologous genes, followed by univariate Cox regression analysis for identifying prognostic CAF-related DEGs based on the clinical data and gene expression data in the GSE116918 dataset. The results showed that 186 CAF-related DEGs were significantly correlated with the BCRFS of prostate cancer patients, and the top 15 significant prognostic CAF-related DEGs were displayed by the Forest plot (Fig. 3A). Meanwhile, 142 CAF-related genes were significantly correlated with the MFS of prostate cancer patients, and the top 15 significant prognostic CAF-related DEGs are shown in Fig. 3B. Further survival analysis was conducted to verify the prognostic value of these genes in prostate cancer patients. The survival curves of the top 3 genes with the significant P value in univariate Cox regression analysis were displayed. As results, high *KCTD4* expression, low *THBS2* expression, and high *ACPP* expression were associated with favorable BCRFS of prostate cancer patients (Fig. 3C–E); and high expression levels of *HOPX*, *TMEM132A*, and *ZNF467* were associated with shorter MFS of prostate cancer patients (Fig. 3F–H). These data were consistent with the results of univariate Cox regression analysis.

### Construction and validation of the prognostic CAF-related gene signatures

**Fig. 4:**
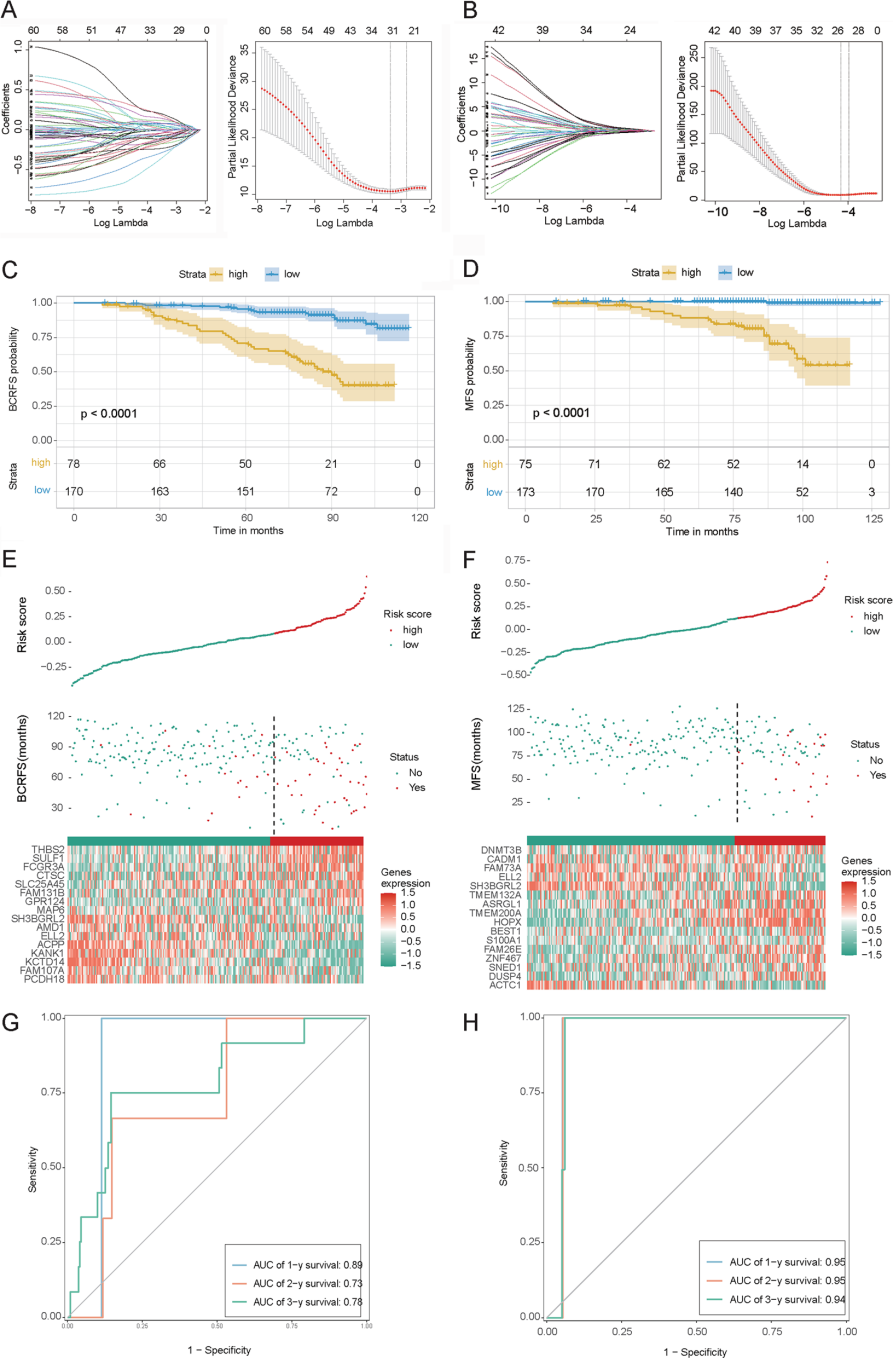
Construction and validation of the prognostic CAF-related gene signatures. **A**, **B** Coefficient profiles of CAF-related DEGs associated with BCRFS or MFS, and the lambda selection in the LASSO model using ten-fold cross-validation. **C**, **D**: Kaplan–Meier survival curve analysis showed the survival of high‐risk and low‐risk patient cohorts divided by BCRFS-related CAF signature or MFS-related CAF signature. **E**, **F** The distribution of the risk score, BCRFS or MFS status as well as the expression levels of genes in the BCRFS-related CAF signature or MFS-related CAF signature. **G**, **H** ROC analysis showed that the AUCs of BCRFS-related CAF signature or MFS-related CAF signature in predicting the 1-, 2-, and 3- year survival of patients with prostate cancer. *CAF* Cancer-associated fibroblast, *DEGs* Differentially expressed genes, *BCRFS* Biochemical recurrence-free survival, *MFS* Biochemical recurrence-free survival, *LASSO*: Least absolute shrinkage and selection operator, *ROC* Receiver operating characteristic, *AUC* Areas under the ROC curve

The prognostic CAF-related gene signatures associated with BCRFS and MFS were then constructed using LASSO Cox regression analysis. The ten-fold cross-validation was employed to determine the final lambda for construction of prognostic CAF-related gene signatures. The results showed that when the lambda was 0.0863, the partial likelihood deviance of the BCRFS-related CAF signature was lowest, indicating that the performance of this model was good (Fig. 4A). A total of 16 CAF-related genes were included in this model, including *AMD1*, *KANK1, ACPP*, *GPR124*, *CTSC*, *SH3BGRL2*, *KCTD14*, *SULF1*, *FCGR3A*, *PCDH18*, *FAM107A*, *ELL2*, *SLC25A45*, *MAP6*, *FAM131B*, and *THBS2*. Meanwhile, we found that the performance of the MFS-related CAF signature was good when lambda was 0.0535 (Fig. 4B), and there were 16 CAF-related genes in this model, including *CADM1*, *SNED1*, *SH3BGRL2*, *HOPX*, *ASRGL1*, *ZNF467*, *TMEM132A*, *ACTC1*, *ELL2*, *FAM73A*, *S100A1*, *BEST1*, *FAM26E*, *TMEM200A*, *DNMT3B*, and DUSP4. We checked the correlation of expression between the identified signature and the apoptosis-related genes and found significant co-expression between them (Additional file 1: Figure S3). To reveal the prognostic value of two CAF signatures, survival analysis was performed. Based on the optimal cut-off for the risk score of each CAF signature, patients were divided into high- and low-risk groups. The prostate cancer patients in the high-risk group all had a significantly poor prognosis (p < 0.0001, Fig. 4C, D). Moreover, prostate cancer patients were ranked according to the risk scores calculated based on the two CAF signatures. The scatter dot plot revealed that the BCRFS or MFS of prostate cancer patients was correlated with the risk score, and patients with a higher risk score were inclined to experience BCR or MET. Meanwhile, the heatmap showed distinct differences in the expression levels of the CAF signature-related genes in the high- and low-risk patients (Fig. 4E, F). Furthermore, receiver operating characteristic (ROC) analysis showed that the areas under the ROC curve (AUCs) of BCRFS-related CAF signature in predicting the 1‐, 2‐, and 3‐year survival of patients with prostate cancer were 0.89, 0.73, and 0.78 (Fig. 4G), and those of MFS-related CAF signature were 0.95, 0.95, and 0.94 (Fig. 4H), indicating the high predictive power of the two CAF signatures.

**Fig. 5:**
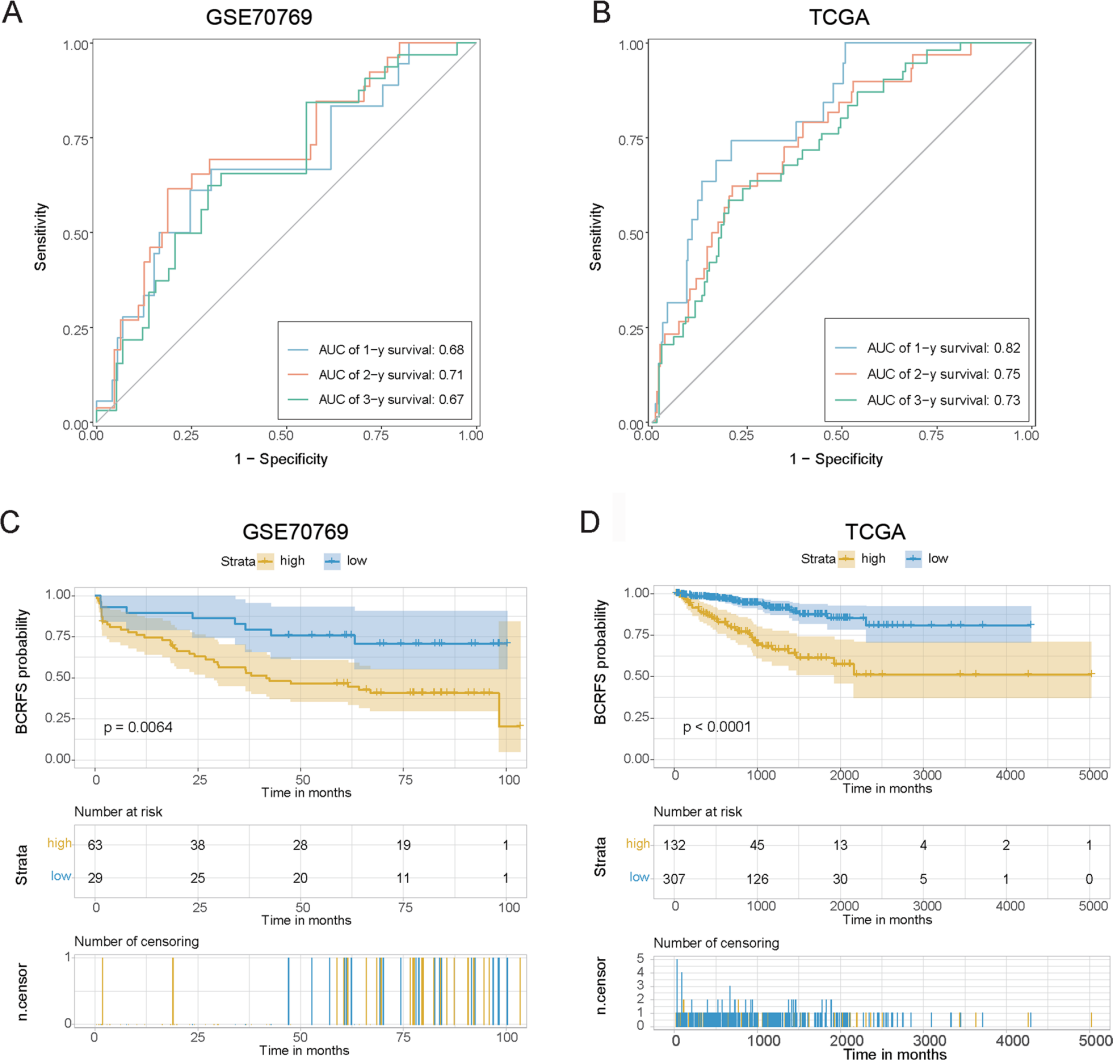
Validation of the prognostic performance of BCRFS-related CAF signature using GSE70769 and TCGA datasets. **A**, **B** ROC analysis showed that the AUCs of BCRFS-related CAF signature in predicting the 1-, 2-, and 3- year survival of patients with prostate cancer based on GSE70769 and TCGA datasets. **C**, **D** Kaplan–Meier survival curve analysis showed the survival of high‐risk and low‐risk patient cohorts divided by BCRFS-related CAF signature based on GSE70769 and TCGA datasets. CAF: cancer-associated fibroblast; BCRFS: biochemical recurrence-free survival; ROC: receiver operating characteristic; AUC: areas under the ROC curve

In addition, we further validate the prognostic performance of BCRFS-related CAF signature using GSE70769 and TCGA datasets. The results showed that the AUCs of BCRFS-related CAF signature in predicting the 1‐, 2‐, and 3‐year survival of patients with prostate cancer were 0.68, 0.71, and 0.67 based on the GSE70769 dataset (Fig. 5A), and those were 0.82, 0.75, and 0.73 based on TCGA dataset (Fig. 5B), confirming the high predictive power of BCRFS-related CAF signature. Also, survival analysis demonstrated that prostate cancer patients with high-risk scores had significantly shorter survival than those with low-risk scores based on GSE70769 (Fig. 5C) and TCGA (Fig. 5D) datasets.

### Correlation analysis of the BCRFS-related CAF signature with various clinical characteristics of patients with prostate cancer

**Fig. 6:**
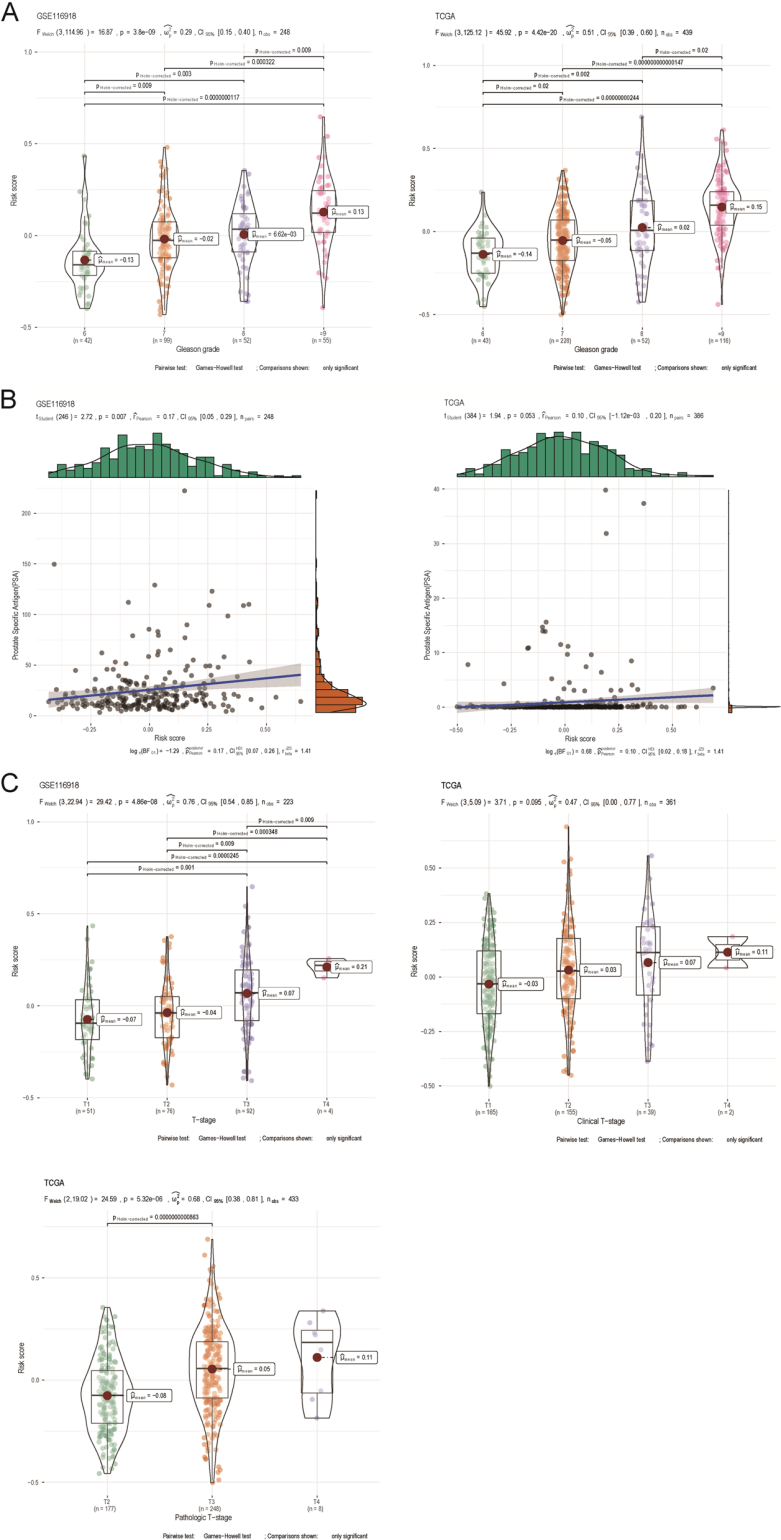
Correlation analysis of the BCRFS-related CAF signature with various clinical characteristics of patients with prostate cancer based on GSE116918 and TCGA datasets. **A** Correlation analysis of risk score with gleason grade. **B** Correlation analysis of risk score with PSA. **C** Correlation analysis of risk score with clinical T stage and pathologic T stage. CAF: cancer-associated fibroblast; BCRFS: biochemical recurrence-free survival; PSA: prostate specific antigen

We further analyzed the correlation between the risk score of the BCRFS-related CAF signature and multiple clinicopathological characteristics of prostate cancer patients based on GSE116918 and TCGA datasets. It was observed that prostate cancer patients with the higher gleason grades showed higher risk scores than those with lower gleason grades based on both GSE116918 and TCGA datasets (Fig. 6A). There was no significant correlation between PSA and risk score (Fig. 6B). In addition, the risk scores were statistically higher in patients with higher clinical T stages than those with lower clinical T stages based on GSE116918 dataset (Fig. 6C). However, the risk scores were similar among different clinical T stages based on TCGA dataset (Fig. 6C). Meanwhile, patients with pathologic T3 stage had higher risk scores than those with pathologic T2 stage (Fig. 6C).

### The BCRFS-related CAF signature was an independent prognostic factor

**Fig. 7:**
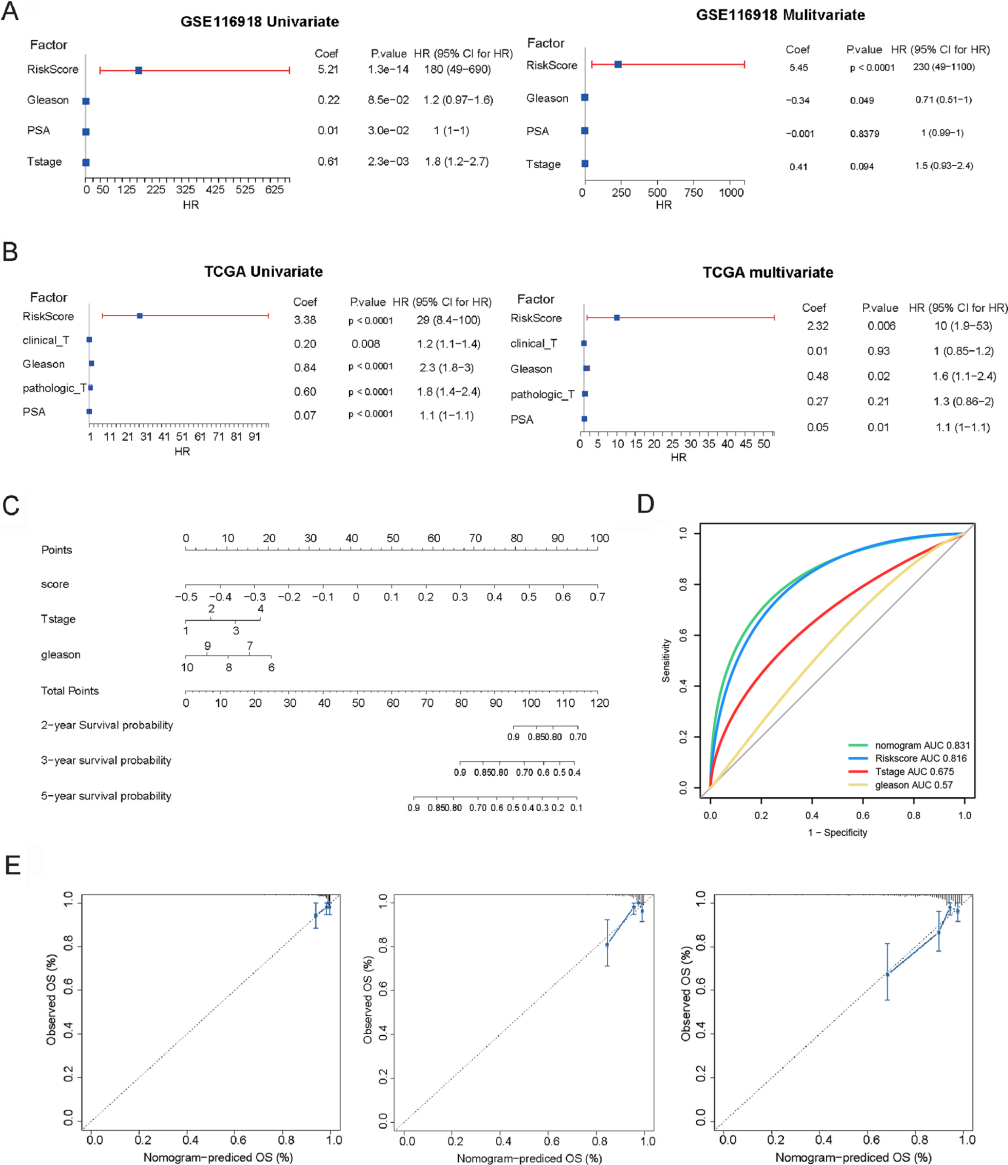
The BCRFS-related CAF signature was an independent prognostic factor and construction and validation of the prognostic nomogram with BCRFS-related CAF signature as one of the parameters. **A** Univariate and multivariate Cox regression analysis shows the correlation of survival with risk score and various clinical parameters based on GSE116918 dataset. **B** Univariate and multivariate Cox regression analysis shows the correlation of survival with risk score and various clinical parameters based on TCGA dataset. **C** The predicted 2-, 3-, 5-year survival rates of prostate cancer patients based on the prognostic nomogram constructed using the risk score and clinicopathological parameters. **D** ROC analysis showed that the AUCs of nomogram, risk score, tumor stage and gleason score. **E** Calibration curves show the concordance between predicted and actual 2-, 3-, and 5-year survival rates of patients. CAF: cancer-associated fibroblast; BCRFS: biochemical recurrence-free survival; ROC: receiver operating characteristic; AUC: areas under the ROC curve

The results of univariate and multivariate Cox regression analyses showed that the risk score calculated from the BCRFS-related CAF signature were significantly associated with survival based on GSE116918 (Fig. 7A) and TCGA (Fig. 7B) datasets (all p < 0.01), indicating that risk score was an independent prognostic factor for prostate cancer patients. Using risk score and other clinicopathological factors, including clinical T stage and gleason grade, a nomogram was established to accurately estimate the 2-, 3-, and 5-year survival probabilities of prostate cancer patients (Fig. 7C). ROC curves showed that the AUC values of nomogram and risk score were 0.831 and 0.816, respectively, indicating that nomogram had a good performance for BCRFS prediction (Fig. 7D). Also, its performance outperformed other clinical variables alone. The calibration curve analysis also showed that the predicted 2-, 3-, and 5-year survival times were consistent with the actual survival times (Fig. 7E). These results demonstrated that the constructed nomogram was reliable and accurate.

### Association analysis of genes in CAF signatures with BCR and MET

**Fig. 8:**
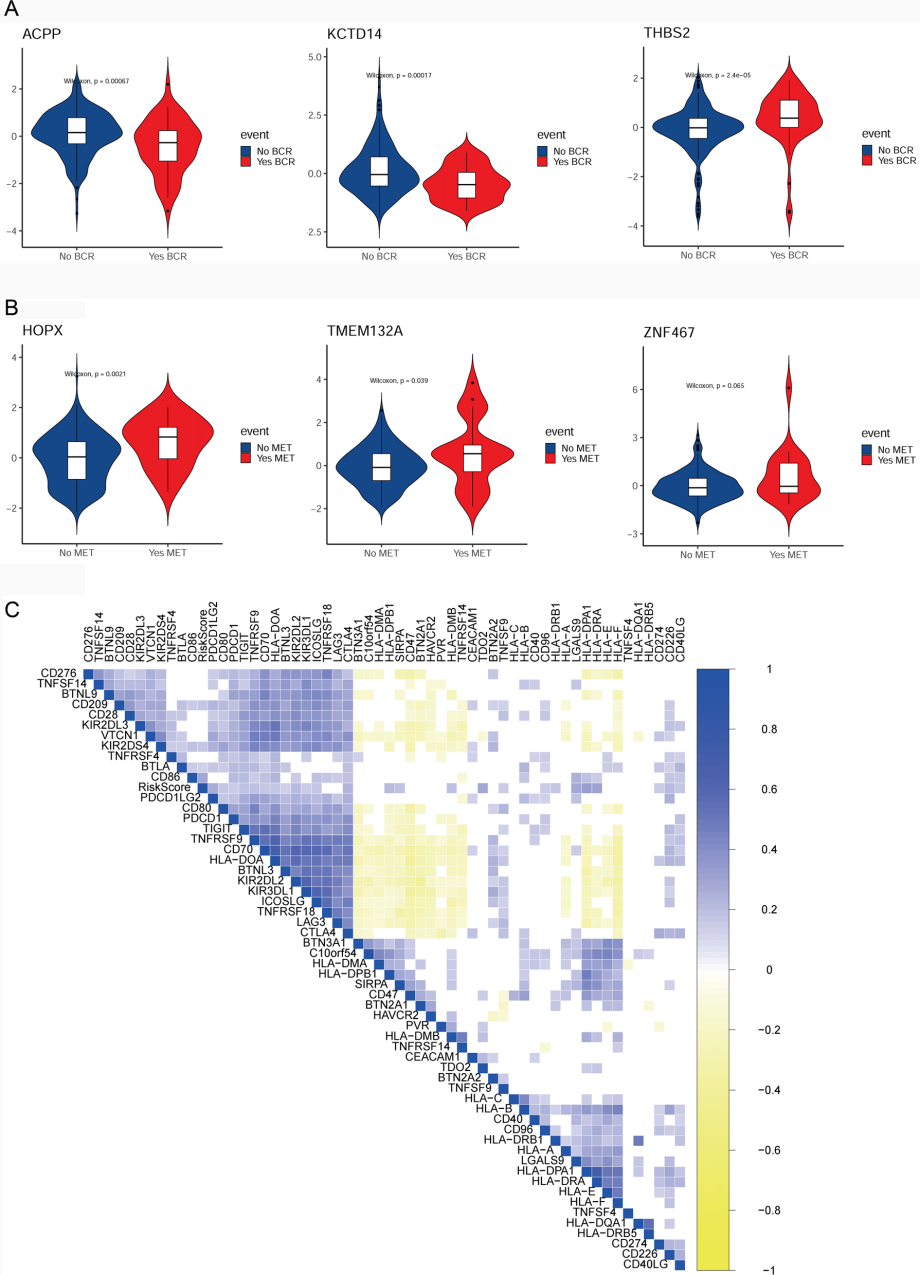
Association analysis of genes in CAF signatures with BCR, MET and immune checkpoints based on the gene expression data in GSE116918 dataset. **A** The expression levels of four randomly selected genes in the BCRFS-related CAF signature, such as *ACPP*, *KCTD14*, and *THBS2* between BCR and non-BCR groups. **B** The expression of four randomly selected genes in the MFS-related CAF signature, such as *HOPX*, *TMEM132A*, *ZNF467*, and *TMEM132A* between MET and non-MET groups. **C** Correlation analysis of BCRFS-related CAF signature with immune checkpoints. *CAF* Cancer-associated fibroblast, *BCR* Biochemical recurrence, *MET* Metastasis, *BCRFS* Biochemical recurrence-free survival, *MFS* Biochemical recurrence-free survival, *PSA* Prostate specific antigen

Based on the gene expression data in GSE116918 dataset, we investigated whether the expression levels of CAF signature genes were associated with BCR or MET. It was observed that there were significant decreased expression of *ACPP* and *KCTD14* and increased expression of *THBS2* between BCR and non-BCR groups (Fig. 8A). Meanwhile, *HOPX* and *TMEM132A* were found to be significantly upregulated in MET group relative to non-MET group, however, there was no significantly difference in *ZNF467* expression between the two groups (Fig. 8B).

### Correlation analysis of BCRFS-related CAF signature with immune checkpoints

To further reveal whether BCRFS-related CAF signature affected patients’ prognosis by modulating immunoregulatory functions, correlation analysis between BCRFS-related CAF signature and immune checkpoints was investigated. The results showed that risk score was strongly positively correlated with multiple immune checkpoints, including PDCD1LG2, CD80, PDCD1, TIGIT, TNFRSF9, CD70, HLA-DOA, BTNL3, KIR2DL2, KIR3DL1, ICOSLG, and TNFRSF18 (Fig. 8C).

## Discussion

Prostate cancer is a public health burden that requires improved patient stratification to accurately predict the risk and treatment response. The risk assessment and management of therapeutic strategies are mainly based on clinical criteria, such as serum PSA, clinical stage, and histopathological features like gleason score [26]. However, these clinical indicators are insufficient to accurately assess disease risk and treatment response [26, 27], emphasizing the urgent need for additional molecular prognostic markers.

Several molecular signatures have been developed to predict the prognosis and treatment response. For instance, an autophagy-related gene expression signature has strong prognostic value in prostate cancer patients [28]. A 28-gene hypoxia signature is a reliable tool for prognosis prediction for prostate cancer patients [29]. A nine-gene expression-based signature could be applied for BCRFS prediction in prostate cancer patients after prostatectomy [30]. These gene signatures can provide information about tumor progression, cancer recurrence, or treatment outcome. Recently, CAFs are recognized as key regulators in the tumorigenesis and metastasis of prostate cancer [31]. A previous study has revealed that prognostic CAF-related signatures can be used as robust prognostic indicators in colon cancer [32] and colorectal cancer [33]. Furthermore, radiotherapy can promote activation of CAFs and thus regulating the effects of the TME on radiotherapy response [34]. In the present study, we identified CAF-related DEGs associated with radiotherapy and established two CAF-related gene signatures for predicting BCRFS and MFS in prostate cancer patients, respectively. The results showed that patients with different risks could be classified by our established prognostic CAF signatures, and patients with higher-risk scores showed shorter BCRFS or MFS times. ROC curve analysis also validated the high prognostic accuracy of the two prognostic CAF signatures. These data revealed that our constructed CAF signatures were reliable and could accurately predict BCRFS and MFS in prostate cancer patients after radiotherapy.

Using univariate Cox regression analysis, 186 CAF-related DEGs were observably correlated with the BCRFS of prostate cancer patients, 16 of which were selected to construct a prognostic CAF signature for predicting BCRFS, such as *ACPP*, *THBS2*, and *KCTD14*. ACPP (prostate acid phosphate) is a secreted glycoprotein enzyme that is produced by in epithelial cells of the prostate gland in humans. ACPP has been reported as a prognostic biochemical indicator for monitoring of prostate cancer progression [35]. ACPP is also shown to promote the osteoblastic reaction in CRPC bone metastases [36]. THBS2 (thrombospondin-2) is a secreted matricellular glycoprotein that is closely related to tumor occurrence and metastasis [37]. It is reported that THBS2 promotes bone metastasis of prostate cancer through inducing miR-376c-mediated MMP2 upregulation [38]. Slavin et al. demonstrated that CAFs inhibit prostate cancer invasion by modulation of the ERα/THBS2/MMP3 axis [14]. However, the role of *KCTD14* (potassium channel tetramerization domain containing 14) has not been investigated. Given the roles of these CAF-related DEGs, our data prompted us to speculate that these CAF-related DEGs might affect the BCR in prostate cancer after radiotherapy. Furthermore, we also identified 142 CAF-related DEGs that were prominently correlated with the MFS of prostate cancer patients, 16 of which were selected to construct a prognostic CAF signature for predicting MFS, such as *HOPX*, *TMEM132A*, and *ZNF467*. *HOPX* (homeodomain only protein X) is identified as a metastasis-associated gene, which downregulation can control metastatic behavior in sarcoma cells [39]. *TMEM132A* (transmembrane protein 132A) is regarded as a novel regulator of Wnt signaling pathway [40], which drives prostate cancer bone metastatic tropism and invasion [41]. ZNF467 (Zinc finger protein 467) is found upregulated in metastatic prostate tumors relative to primary tumors [42]. Fan et al. indicated that a gene signature involving three enhancer RNAs-driven genes including *ZNF467* was a good predictor of the prognosis of prostate cancer patients [43]. These data suggested the potential role of these CAF-related DEGs in regulating tumor metastasis in prostate cancer.

Strikingly, a nomogram has been applied as an effective and reliable clinical tool for evaluating the survival of cancer patients [44]. Moreover, the combination of the serum marker PSA, clinical stage, and gleason score of the prostate biopsy is currently used to stratify patients into different risk groups for biochemical recurrence [45]. Therefore, we developed a robust nomogram consisting of the risk scores based on the BCRFS-related CAF signature and several clinical variables (PSA, gleason score and clinical T stage) to improve prognostic prediction of prostate cancer patients. We found that the AUC values of nomogram and risk score were 0.831 and 0.816, respectively, and calibration plots displayed that the actual and predicted 1-, 3-, and 5-year survival rates based on the nomogram were similar. As there were limited validation prostate cancer cohorts who received the radiotherapy, we used the overall survival as the surrogate of radiotherapy response. In general, the higher the degree of malignancy, the worse the response to radiotherapy as well as the overall survival. We expected further available data sets to complement the validation. Taken together, we believed that our constructed nomogram showed great potential for clinical applications for prostate cancer patients, such as individualized treatment and prognosis.

To better understand the function of CAF-related DEGs associated with radiotherapy, we conducted functional enrichment analysis and found that human homologous genes of these DEGs were significantly enriched ECM-related functions, such as extracellular matrix structural constituent and cell adhesion molecules. Increasing evidence has revealed that ECM remodeling play a crucial role in tumor progression and metastasis [45] as well as cancer cell survival [46]. ECM remodeling is also shown to alter tumor microenvironment and mediates tumor progression and resistance to therapy [47]. CAFs can deposit ECM components and regulate migration and invasion of cancer cells via modulating remodeling of the microenvironment [48, 49]. Overall, our data implied that CAF-related DEGs might regulate radiotherapy response in prostate cancer through regulating ECM remodeling. Furthermore, CAFs are recognized contributors of tumor immune evasion [50]. CAFs can affect the anti-tumor immune response by influencing the recruitment of immune cells and driving an immunosuppressive function in immune cells [51, 52]. Accumulating evidence has revealed that CAFs are implicated in the induction of radioresistance, and the crosstalk between CAFs, tumor cells, and immune cells affects radiotherapy outcome [53, 54]. Herein, we found that the CAF-related DEGs were markedly enriched in immune-related functions, such as regulation of leukocyte migration and the risk scores were positively correlated with multiple immune checkpoints. Therefore, we speculated that CAF-related DEGs might affect radiotherapy outcome for prostate cancer regulating immune response.

## Conclusion

Our study for the first time successfully establishes a cell subline resistant to irradiation (CAFR), which is more radio-resistant to irradiation than the parental cell line CAF. Moreover, based on the CAF-related genes by analysis of transcriptome sequencing data, our constructed CAF signatures could accurately predict BCRFS and MFS in prostate cancer patients undergoing radiotherapy. The nomogram constructed by the prognostic CAF signature and other clinical features facilitates an individualized and accurate BCRFS prediction. CAF-related DEGs might regulate radiotherapy outcomes in prostate cancer through modulating ECM remodeling and immune response.

## Supplementary Information

**Additional file 1: Figure S1.** The clones of the CAF and CAFR group after 0, 4, 8 and 10 Gy irradiation. Representative dishes after colony-forming assay are shown. **Figure S2.** The senescence and proliferative capacity of CAF and CAFR. (**A**) Radiation-induced senescence was detected by a β-galactosidase assay. Blue color, β-gal positive cells. Percentage of β-galactosidase positive cells in each group was calculated and showed in bottom. (**B**) Cell proliferation curve detected by MTT. **Figure S3.** Co-expression analysis (spearman correlation) between signature and the apoptosisrelated genes.

## Data Availability

The data used to support the findings of this study are available from the corresponding author upon request.
