# Supplementary material for: Cancer-associated fibroblast-derived gene signatures predict radiotherapeutic survival in prostate cancer patients
Source: J Transl Med. 2022 Oct 4;20:453. doi: 10.1186/s12967-022-03656-5 (PMC9533530; doi:10.1186/s12967-022-03656-5)
Supplement: Supplementary file 1 — Additional file 1: Figure S1. The clones of the CAF and CAFR group after 0, 4, 8 and 10 Gy irradiation. Representative dishes after colony-forming assay are shown. Figure S2. The senescence and proliferative capacity of CAF and CAFR. (A) Radiation-induced senescence was detected by a β-galactosidase assay. Blue color, β-gal positive cells. Percentage of β-galactosidase positive cells in each group was calculated and showed in bottom. (B) Cell proliferation curve detected by MTT. Figure S3. Co-expression analysis (spearman correlation) between signature and the apoptosisrelated genes. [file 12967_2022_3656_MOESM1_ESM.pdf]

Supplementary Materials

Cancer-associated fibroblast-derived gene signatures predict radiotherapeutic survival in prostate cancer patients

Ran Zhang[1], Feng Liu[2, *]

1. Laboratory of Radio-Immunology, Shandong Provincial Key Laboratory of Radiation Oncology, Cancer Research Center, Shandong Cancer Hospital and Institute, Shandong First Medical University and Shandong Academy of Medical Sciences, Jinan, Shandong, 250117, P.R. China

2.Department of Immunology, School of Biomedical Sciences, Shandong University, Jinan, Shandong 250012, P.R. China

* Correspondence to: Dr. Feng Liu, E-mail: liufeng2019@sdu.edu.cn

Fig.S1

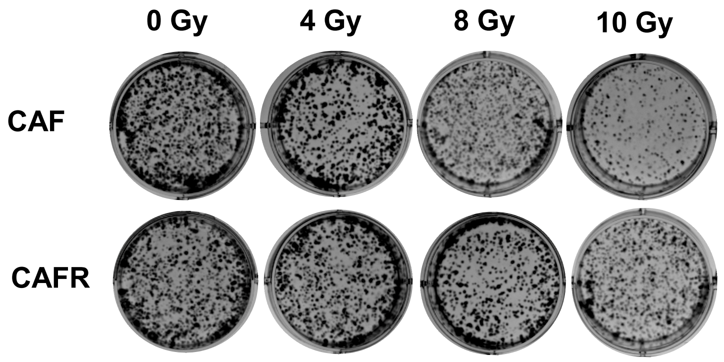

Figure S1. The clones of the CAF and CAFR group after 0, 4, 8 and 10 Gy irradiation. Representative dishes after colony-forming assay are shown.

Fig.12

A.

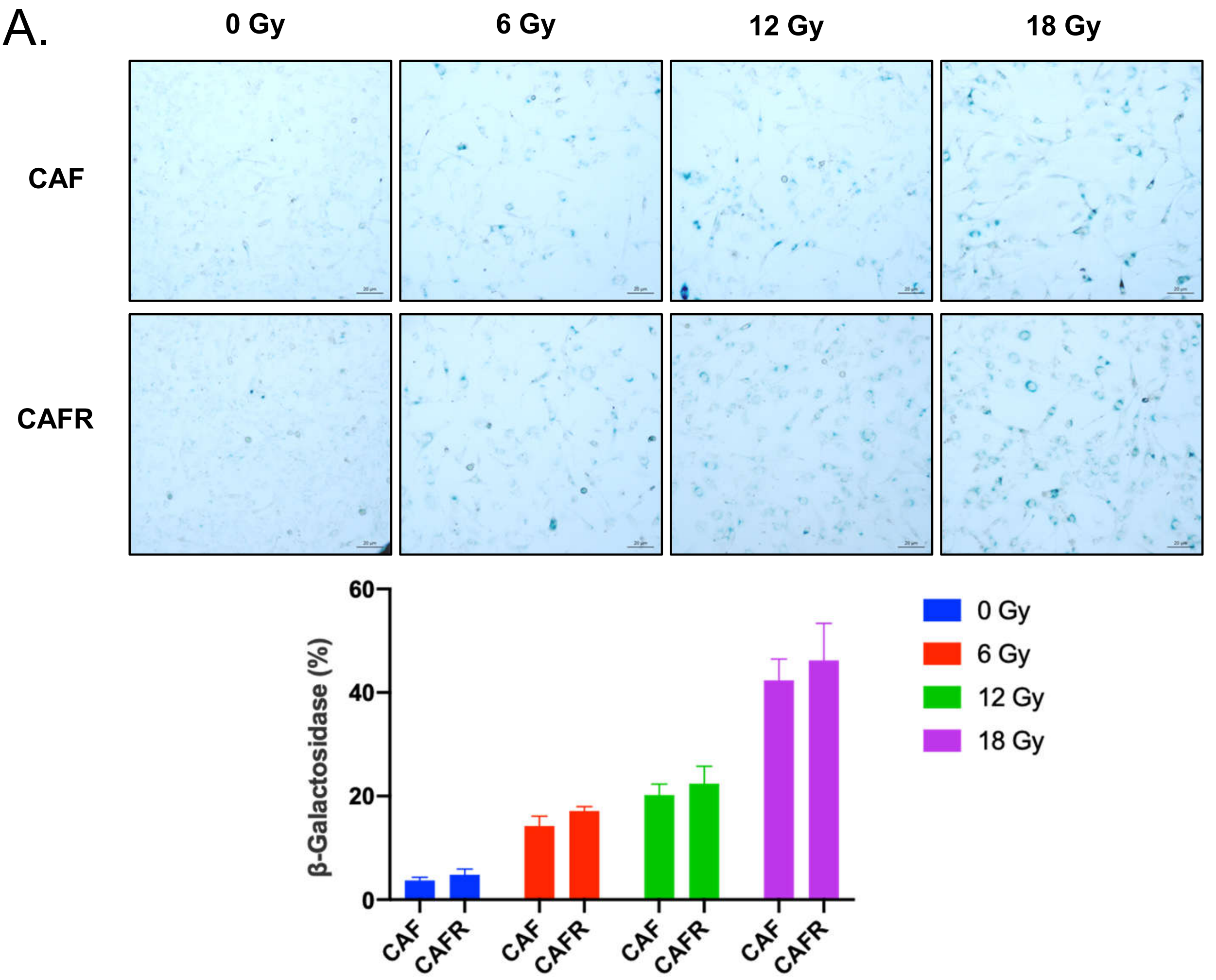

B.

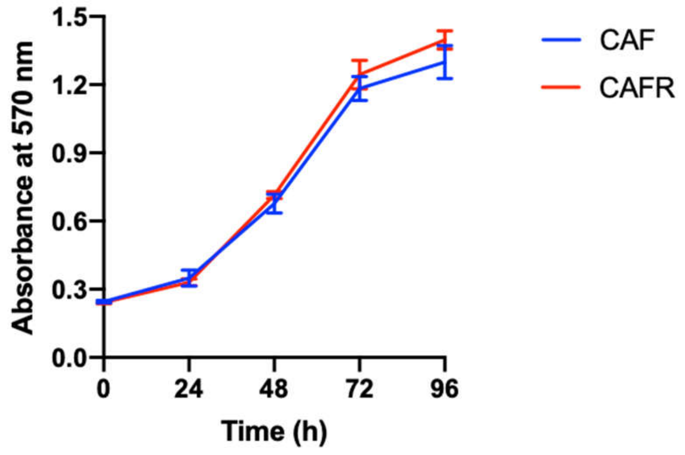

Figure S2. The senescence and proliferative capacity of CAF and CAFR.

(A) Radiation-induced senescence was detected by a β-galactosidase assay. Blue color, β-gal positive cells. Percentage of β-galactosidase positive cells in each group was calculated and showed in bottom.

(B) Cell proliferation curve detected by MTT.

Fig.S3

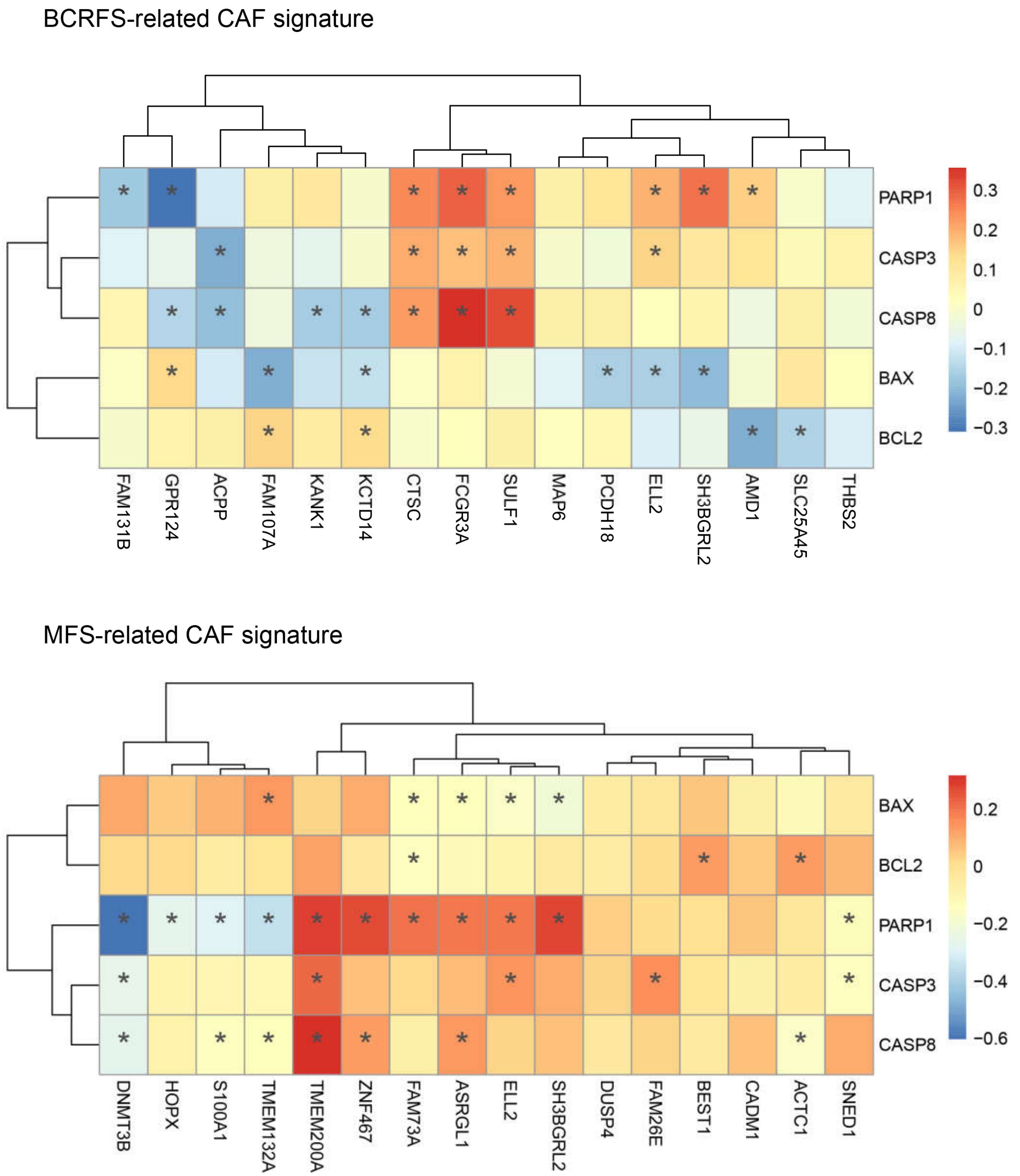

Figure S3. Co-expression analysis (spearman correlation) between signature and the apoptosis-related genes.
